# Association of adiponectin gene variants with idiopathic recurrent miscarriage according to obesity status: a case–control study

**DOI:** 10.1186/s12967-018-1453-3

**Published:** 2018-03-20

**Authors:** Maryam Dendana, Wael Bahia, Ramzi R. Finan, Mariam Al-Mutawa, Wassim Y. Almawi

**Affiliations:** 1 Faculty of Pharmacy, University of Monastir, Monastir, Tunisia; 2 University of Carthage, Bizerte, Tunisia; 3 Department of Obstetrics and Gynecology, Hôtel Dieu de France, CHU Université St. Joseph, Beirut, Lebanon; 4 Faculty of Sciences, El Manar University, University Campus, Manar II, 2092 Tunis, Tunisia; 5 School of Pharmacy, Lebanese American University, Byblos, Lebanon

**Keywords:** Adiponectin, Haplotypes, Idiopathic recurrent miscarriage, Real-time PCR

## Abstract

**Background:**

This study addresses whether the association of adiponectin gene (*ADIPOQ*) variants with idiopathic recurrent pregnancy loss (RPL) is influenced by obesity.

**Methods:**

Retrospective case–control study performed in outpatient obstetrics/gynecology clinics. Study subjects comprised 308 women with RPL, defined as ≥ 3 consecutive miscarriages of unknown etiology, and 310 control women. *ADIPOQ* genotyping was done by allele exclusion method on real-time PCR.

**Results:**

Of the 14 *ADIPOQ* variants tested, the minor allele frequency (MAF) of rs4632532, rs17300539, rs266729, rs182052, rs16861209, and rs7649121 were significantly higher, while rs2241767, and rs1063539 MAF were lower in RPL cases, hence assigning RPL-susceptibility and protection to these variants, respectively. Higher frequencies of heterozygous rs17300539 and rs16861209, and homozygous rs4632532, rs266729, and rs182052 genotypes, and reduced frequencies of heterozygous rs1063539 and rs2241767, homozygous rs2241766 genotypes were seen in RPL cases. *ADIPOQ* rs4632532, and rs2241766 were associated with RPL in obese, while rs1063539 and rs16861209 were associated with RPL in non-obese women; rs182052 and rs7649121 associated with RPL independently of BMI changes. Based on LD pattern, two haplotype blocks were identified. Within Block 1 containing rs4632532, rs16861194, rs17300539, rs266729, rs182052, rs16861209, rs822396, and rs7649121, increased frequency of CAGGACAT and TAACGAAA, and reduced frequency of TAGCGCAA haplotypes were seen in RPL cases when compared to controls, thereby assigning RPL susceptibility and protection, respectively.

**Conclusion:**

This is the first study to document contribution of *ADIPOQ* variants and haplotypes with RPL, and also to underscore the contribution of obesity to genetic association studies.

## Background

Recurrent pregnancy loss (RPL), defined as ≥ 2 idiopathic miscarriages with the same partner prior the 20th week of gestation [1], is a common pregnancy complication which affects ~ 1–2% of women worldwide [2]. Modifiable and non-modifiable factors that contribute to RPL pathogenesis were reported. The former includes environmental and lifestyle factors (smoking, obesity), poorly-controlled diabetes, hypothyroidism, and infections [3, 4], while the latter includes chromosomal anomalies, age at first pregnancy, and presence of specific genetic polymorphisms [2, 5]. Despite the identification of several risk factors of RPL, the etiology of idiopathic RPL remains not completely understood, with more than 50% of RPL cases still remain unexplained [1, 2].

Normal human pregnancy is linked with altered metabolism stemming from the accumulation of white adipose tissues [6], leading to progressive decrease in insulin sensitivity [6, 7]. White adipose tissues synthetize an array of hormones within the intra-uterine compartment, which serve both metabolic, anti-atherosclerotic, and anti-inflammatory roles. These include leptin, resistin, apelin, and adiponectin [8]. Adiponectin is a 30 kDa, 244 amino acid adipokine, which regulates insulin sensitivity, blood glucose levels, and lipid metabolism, and possesses anti-atherosclerosis and anti-inflammatory activities [9].

Adiponectin is produced mostly from adipocyte, but is also secreted by other tissues, including reproductive organs [9]. Adiponectin is detected in amniotic fluid after 15 weeks of pregnancy, and its maternal plasma concentrations remain virtually unchanged throughout pregnancy [10, 11]. Reduction in adiponectin levels results in reproductive disorders [12, 13], including failed embryo implantation, polycystic ovarian syndrome (PCOS), endometriosis, gestational diabetes mellitus (GDM) [14, 15], and RPL [16]. Adiponectin is encoded by *ADIPOQ* gene (adipocyte C1Q and collagen domain), located on chromosome 3q27 spanning 17 kB, and containing 3 exons and two introns [17], and is highly polymorphic. Previous studies demonstrated association of functional *ADIPOQ* gene variants with altered circulating adiponectin concentrations [18, 19], and with pregnancy complications, including GDM, metabolic syndrome, and preeclampsia (PE) [20–22]. Decreased adiponectin serum levels were also linked with severe obesity [23], endometriosis and PCOS [12].

Very few studies examined the association of *ADIPOQ* variants with idiopathic RPL. An earlier Indian study documented the association of *ADIPOQ* rs2241766 (exon 2) and rs15012299 (intron 2) with increased risk of RPL [16]. In addition, *ADIPOQ* rs2241766 and rs15012299 variants showed weak, but statistically significant, haplotype association with PE susceptibility in Finnish women [24]. Here we analyze the association of 14 common *ADIPOQ* variants in women with confirmed RPL diagnosis, and multiparous control women who were matched to RPL cases according to self-declared racial background. The contribution of these variants to RPL was examined according to obesity, and was examined at the allele, genotype, and haplotype levels.

## Subjects and methods

### Subjects

This retrospective case–control study was performed between January 2013 and June 2015. Study population included 308 women with confirmed RPL who were recruited consecutively from the outpatient OB/GYN clinics in Manama and Riffa (Bahrain). In addition, we recruited 310 multiparous women with two or more successful pregnancies as controls. The study protocol was approved by Arabian Gulf University Research and Ethics Committee (IRB approval: 35-PI-01/15), and was done according to Helsinki II Declaration. All patients provided informed written consent before blood sampling.

We defined RPL as ≥ 3 consecutive pregnancy losses of undetermined etiology, which occurred between the 7th–20th week of gestation, and with the same partner. RPL assessment was according to the guidelines of the Royal College of Obstetricians and Gynecologists (https://www.rcog.org.uk/guidelines). These required screening for LAC (lupus anticoagulant) and ACL (anticardiolipin) anti-phospholipid antibodies, and the inherited thrombophilias Factor V-Leiden (R506Q) and factor II/prothrombin G20210A. Karyotyping of both parents, and pelvic ultrasound scan by hysteroscopy or sonohysteroscopy for evaluation of uterine anatomy were also performed on all RPL cases. RPL cases were excluded if they were 40 years or older at first pregnancy, incompatibility in Rh blood groups, history of PE, which was defined as elevation in systolic/diastolic blood pressure (BP) exceeding 145/95 mmHg, or increase in systolic/diastolic BP exceeding 30/15 mmHg on at least two occasions, as well as biochemical pregnancy, and preclinical miscarriages. Cases were also excluded if they had systemic autoimmunity, thyroid dysfunction, diabetes, liver function abnormalities, anatomical disorders, and infections (toxoplasmosis, HIV, HCMV, Group B streptococci, *Chlamydia trachomatis*, hepatitis B and C viruses, rubella, and bacterial vaginosis).

Control women comprised hospital and university students and employees, and volunteers from the community, and were included if they had two or more successful pregnancies. Control women were included following check-up after uncomplicated pregnancy/delivery. Controls were excluded if they reported spontaneous and/or induced miscarriages, and family history of miscarriage, and were matched to RPL cases according to age (*P *= 0.94), and self-reported ethnic origin (all Bahraini Arabs). Peripheral blood samples (2–5 ml) were collected from RPL cases and control women in EDTA-containing tubes for DNA extraction.

### *ADIPOQ* genotyping

*ADIPOQ* single nucleotide polymorphisms (SNP) with minor allele frequencies (MAF) that exceeded 5% in Caucasians (HapMap CEU), were selected using SNPbrowser software 4.0 (ABI-ThermoFisher, Foster City, CA). Genotyping was done by the allelic discrimination method using VIC-/FAM-labelled primers (Table 2). Assay-on-demand TaqMan assays were obtained from ABI-ThermoFisher. PCR was performed in 6 μl volume on StepOne Plus real-time PCR system, according to instructions of the manufacturer (ABI-ThermoFisher). A typical RT-PCR consisted of adding 2.2 µl DNA template, and 4.0 µl TaqMan genotype master mix (TaqMan 2X mix, 1.875 µl nuclease free water, and 0.125 µl 40X SNP primer mix) (Applied Biosystem). Pre-PCR (hold step) stage was performed for 30 s at 60 °C, and for 10 min at 95 °C. The cycles conditions will be repeated 35 times, and consisted of denaturation (92 °C for 15 s), annealing and extension (60 °C for 1 min), followed by post-PCR stage at 60 °C for 30 s. Replicate quality control samples (≈ 10% of cases and controls) were included for assessment of genotyping reproducibility; concordance consistently exceeded 99%.

### Statistical analysis

Statistical analysis was done on SPSS v. 24 (IBM, Armonk, NY). Categorical variables were expressed as percent of total, while continuous data were presented as mean ± SD. Differences in means were analyzed using Student’s t test, and inter–group significance was assessed by Pearson χ^2^ or Fisher’s exact test. Hardy–Weinberg equilibrium (HWE) calculation was done for control women using Haploview 4.2 (https://www.broad.mit.edu/mpg/haploview). SNPStats (https://bioinfo.iconcologia.net/snpstats) was used for genetic association analysis, under the assumption of additive genetic model. Power calculation for detection of association between *ADIPOQ* variants and RPL was done using CaTS Power Calculator (https://www.sph.umich.edu/csg/abecasis/cats). We used the following parameters: 308 RPL cases and 310 control women, relative risk for heterozygous and minor allele homozygous genotypes, and MAF for the 14 tested *ADIPOQ* SNPs RPL in cases and controls, and assuming a 2.5% population RPL prevalence in Bahrain (Bahrain Ministry of Health unpublished statistics). The overall power was calculated as 72.1%, which represented the average power of the included SNPs. Haploview 4.2 was used for determination of linkage disequilibrium (LD) and haplotype reconstruction, the latter done according to expectation maximization (EM) algorithm. Taking control women as the reference group, logistic regression analysis was used in determining odds ratios (OR) and 95% confidence intervals (CI) associated with the risk of RPL; *P *< 0.05 was taken as statistically significant.

## Results

### Demographic and clinical characteristics of RPL cases and control women

Table 1 summarizes the demographic and clinical profiles of RPL cases and control women. Age at entry into the study, fasting plasma glucose, gravida, and number of smokers were not significantly different between women with RPL and control subjects. Significant differences between the two study groups were seen in mean BMI (*P *= 0.004), menarche (*P *< 0.001), and in systolic/diastolic blood pressure readings (*P *< 0.001). While these did not constitute established RPL risk factors, we nevertheless included them as covariates which we controlled for in later analysis.

**Table 1:** Demographics and clinical characteristics of cases and controls

	Cases^a^	Controls^a^	*P* ^b^
Age at inclusion in study^c^	31.6 ± 5.4	31.6 ± 4.9	0.94
Body-mass index (kg/m^2^)^c^	26.3 ± 5.4	25.2 ± 4.3	0.004
Obesity [n (%)]^d^	58 (19.6)	37 (12.1)	0.02
Smokers [n (%)]^d^	30 (10.1)	32 (10.8)	0.69
Systolic blood pressure (mmHg)^c^	114.2 ± 11.9	120.2 ± 17.0	< 0.001
Diastolic blood pressure (mmHg)^c^	72.0 ± 8.4	75.8 ± 9.1	< 0.001
Glucose (mmol/L)^c^	5.1 ± 0.9	5.2 ± 0.7	0.55
Menarche (years)^c^	12.2 ± 1.1	12.8 ± 1.0	< 0.001
Number of pregnancies^c^	4.2 ± 1.5	4.0 ± 1.1	0.11
Number of children^c^	0.8 ± 1.1	4.0 ± 1.1	< 0.001
Miscarriages^c^	3.6 ± 1.0	0.0 ± 0.1	< 0.001

^a^A total of 308 RPL cases and 310 control women were included

^b^Student’s *t*-test (continuous variables), Pearson’s χ^2^ test (categorical variables)

^c^Mean ± SD

^d^Percent of total within each group/subgroup

### Association between *ADIPOQ* SNP and the risk of RPL

Table 2 presents the association between *ADIPOQ* SNP and RPL in women with RPL and control women. Genotype distributions of all 14 tested *ADIPOQ* variants conformed to HWE among study subjects. Among *ADIPOQ* SNP tested, higher MAF of rs4632532 (*P* = 8.00 × 10^−3^), rs17300539 (*P *= 0.011), rs266729 (*P *= 4.00 × 10^−3^), rs182052 (*P* = 7.00 × 10^−4^), rs16861209 (*P* = 9.00 × 10^−3^), rs7649121 (*P *= 0.044), thereby assigning RPL susceptibility to these variants. On the other hand, MAF of rs2241767 (*P* = 0.041) and rs1063539 (*P* = 0.048), was lower in women with RPL than in control women, suggesting RPL-protection associated with these *ADIPOQ* variants. No significant differences in MAF of the remaining SNPs were seen between women with RPL and controls.

**Table 2:** *ADIPOQ* SNPs analyzed in RPL cases and control women

SNP	Assay ID	Position	HWE P	Alleles	Cases^a^	Controls^a^	χ^2^	*P*	aOR^b^ (95% CI)	Power
*rs4632532*	C_27867233_10	186551682	0.209	T:C	*0.351*	*0.271*	*7.035*	*0.008*	*1.46 (1.10–1.93)*	*90*
rs16861194	C_33187775_10	186559425	0.325	A:G	0.098	0.079	0.979	0.322	1.25 (0.80–1.95)	63
*rs17300539*	C_33187774_10	186559460	1.000	G:A	*0.087*	*0.044*	*6.534*	*0.011*	*2.06 (1.17–3.63)*	*100*
*rs266729*	C_2412786_10	186559474	0.213	C:G	*0.262*	*0.173*	*8.295*	*0.004*	*1.69 (1.18–2.42)*	*83*
*rs182052*	C_2412785_10	186560782	0.348	G:A	*0.360*	*0.249*	*11.381*	*7.00 × 10* ^*−4*^	*1.70 (1.25–2.31)*	*100*
*rs16861209*	C_33187764_10	186563114	0.205	C:A	*0.144*	*0.087*	*6.852*	*0.009*	*1.77 (1.15–2.72)*	*89*
rs822396	C_2910316_10	186566877	0.337	A:G	0.139	0.157	0.582	0.445	0.92 (0.64–1.33)	42
*rs7649121*	C_42772949_10	186568785	0.303	A:T	*0.182*	*0.134*	*4.063*	*0.044*	*1.44 (1.01–2.05)*	
rs2241766	C_26426077_10	186570892	0.578	T:G	0.156	0.193	2.166	0.141	0.80 (0.57–1.12)	62
rs1501299	C_7497299_10	186571123	0.212	C:A	0.324	0.307	0.343	0.558	1.09 (0.83–1.43)	54
*rs2241767*	C_26426076_10	186571196	1.000	A:G	*0.139*	*0.188*	*4.161*	*0.041*	*0.70 (0.49–0.98)*	*66*
rs3774261	C_27479710_10	186571559	0.303	G:A	0.462	0.495	0.989	0.320	0.87 (0.67–1.14)	61
rs6773957	C_1486294_10	186573705	0.285	G:A	0.506	0.475	0.951	0.330	1.13 (0.88–1.46)	63
*rs1063539*	C_1486290_10	186575392	0.277	G:C	*0.147*	*0.196*	*3.898*	*0.048*	*0.70 (0.50–0.99)*	*68*

Italicface indicates statistical significance

^a^MAF frequency

^b^aOR = adjusted OR; variables that were controlled for were BMI, menarche, systolic and diastolic blood pressure

Table 3 lists the distribution of *ADIPOQ* genotypes between women with RPL and control women. Taking major allele homozygous genotype (1/1) as the reference group (OR = 1.00), after controlling for BMI, systolic/diastolic blood pressure, and menarche, significantly higher frequencies of heterozygous rs17300539 (0.17 vs. 0.08) and rs16861209 (0.32 vs. 0.15), and homozygous rs4632532 (0.13 vs. 0.08), rs266729 (0.11 vs. 0.05), and rs182052 (0.16 vs. 0.05) genotypes were seen in RPL cases vs. control women. In addition, reduced genotype frequencies of heterozygous rs1063539 (0.21 vs. 0.31) and rs2241767 (0.22 vs. 0.31), and homozygous rs2241766 (0.02 vs. 0.07) were seen in women with RPL compared to controls. The distribution of the remaining genotypes was not significantly different between women with RPL and control subjects.

**Table 3:** *ADIPOQ* genotype frequencies

SNP	1/1^a^			1/2^a^			2/2^a^		
	Cases	Controls	*P* ^b^	Cases	Controls	OR (95% CI)	Cases	Controls	OR (95% CI)
rs4632532	141 (0.46)^c^	171 (0.55)	0.042	126 (0.41)	114 (0.37)	1.33 (0.92–1.93)	*41 (0.13)*	*25 (0.08)*	*2.03 (1.11–3.70)*
rs1063539	229 (0.74)	199 (0.64)	0.060	*68 (0.21)*	*95 (0.31)*	*0.63 (0.41–0.96)*	11 (0.04)	16 (0.05)	0.57 (0.23–1.44)
rs17300539	256 (0.83)	283 (0.91)	0.027	*51 (0.17)*	*25 (0.08)*	*2.22 (1.22–4.04)*	1 (0.003)	1 (0.003)	1.08 (0.07–17.41)
rs16861209	229 (0.74)	259 (0.84)	0.032	*69 (0.32)*	*48 (0.15)*	*1.64 (1.01–2.68)*	10 (0.03)	3 (0.01)	3.87 (0.79–18.88)
rs266729	187 (0.61)	208 (0.67)	0.079	88 (0.29)	86 (0.28)	1.14 (0.74–1.77)	*33 (0.11)*	*16 (0.05)*	*2.37 (1.09–5.18)*
rs822396	233 (0.76)	223 (0.72)	0.650	66 (0.21)	78 (0.25)	0.81 (0.53–1.25)	9 (0.03)	9 (0.03)	0.96 (0.33–2.80)
rs1501299	144 (0.47)	161 (0.52)	0.511	127 (0.41)	115 (0.37)	1.23 (0.85–1.79)	37 (0.12)	34 (0.11)	1.23 (0.69–2.17)
rs182052	132 (0.43)	174 (0.56)	0.001	128 (0.42)	119 (0.38)	1.43 (0.94–2.17)	*48 (0.16)*	*17 (0.05)*	*3.67 (1.73–7.76)*
rs7649121	222 (0.72)	244 (0.79)	0.190	57 (0.19)	49 (0.16)	1.30 (0.80–2.10)	29 (0.09)	17 (0.05)	1.76 (0.86–3.58)
rs2241766	219 (0.71)	195 (0.63)	0.043	82 (0.27)	96 (0.31)	0.77 (0.52–1.13)	*7 (0.02)*	*22 (0.07)*	*0.34 (0.13–0.90)*
rs3774261	92 (0.30)	93 (0.30)	0.212	148 (0.48)	127 (0.41)	1.19 (0.76–1.86)	68 (0.22)	90 (0.29)	0.78 (0.47–1.30)
rs16861194	257 (0.83)	264 (0.85)	0.687	46 (0.15)	44 (0.14)	1.09 (0.67–1.76)	5 (0.02)	2 (0.01)	2.01 (0.36–11.08)
rs6773957	80 (0.26)	88 (0.28)	0.790	144 (0.47)	144 (0.46)	1.11 (0.73–1.69)	84 (0.27)	78 (0.25)	1.18 (0.73–1.90)
rs2241767	223 (0.72)	200 (0.65)	0.054	*67 (0.22)*	*96 (0.31)*	*0.62 (0.42–0.93)*	18 (0.06)	14 (0.05)	1.14 (0.51–2.56)

Italicface indicates statistical significance

^a^Genotypes were coded as per “1” = major allele, “2” = minor allele

^b^2-way ANOVA

^c^Number of subjects (frequency)

### Association of *ADIPOQ* polymorphisms with RPL in obese and non-obese RPL subjects

In view of the effect of *ADIPOQ* SNPs on adiponectin secretion, and the impact of adiposity of pregnancy outcome, we examined the association of the tested *ADIPOQ* polymorphisms with RPL in obese and non-obese RPL cases and controls. Women with RPL and control women were sub grouped into non-obese and obese according to BMI cutoff of 30 kg/m^2^. Results from Table 4 demonstrate differential association of *ADIPOQ* SNPs with RPL according to obesity was seen, with rs16861209 (*P *= 0.034), rs182052 (*P *= 0.004), and rs7649121 (*P *= 0.040) being positively associated, while rs1063539 (*P *= 0.028) was negatively associated with RPL in obese subjects (Table 3). In contrast, rs182052 (*P *= 0.005), and rs7649121 (*P *= 0.046) were positively, while rs4632532 (*P *= 0.002), and rs2241766 (*P *= 0.021) were negatively associated with RPL in obese subjects.

**Table 4:** Association of *ADIPOQ* variants with RPL in obese vs. non-obese cases and control women

SNP	Non-obese				Obese			
	Control^a^	Case^a^	*P*	OR (95% CI)	Control^a^	Case^a^	*P*	OR (95% CI)
rs4632532	28.05	33.33	0.093	1.28 (0.96–1.71)	*12.00*	*36.17*	*0.002*	*4.15 (1.60–10.76)*
rs1063539	*20.45*	*14.40*	*0.028*	*0.65 (0.44–0.95)*	23.08	15.85	0.296	0.63 (0.26–1.51)
rs17300539	03.72	06.91	0.053	1.92 (0.98–3.75)	09.26	17.11	0.202	2.02 (0.68–6.06)
rs16861209	*07.88*	*12.64*	*0.034*	*1.69 (1.03–2.76)*	13.46	23.68	0.152	1.99 (0.77–5.19)
rs266729	19.17	24.00	0.117	1.33 (0.93–1.91)	16.67	30.26	0.125	2.17 (0.79–5.92)
rs822396	16.09	14.89	0.647	0.91 (0.62–1.35)	13.79	08.75	0.348	0.60 (0.20–1.76)
rs1501299	29.01	31.86	0.371	1.14 (.085–1.54)	32.14	35.56	0.671	1.16 (0.57–2.36)
rs182052	*26.90*	*37.50*	*0.004*	*1.63 (1.17–2.27)*	*10.71*	*31.43*	*0.005*	*3.82 (1.42–10.24)*
rs7649121	*13.78*	*19.19*	*0.040*	*1.49 (1.02–2.18)*	*10.71*	*26.09*	*0.046*	*2.60 (0.99–6.80)*
rs2241766	20.62	16.09	0.093	0.74 (0.52–1.05)	*30.00*	*14.44*	*0.021*	*0.39 (0.18–0.88)*
rs3774261	32.08	45.45	0.386	0.88 (0.65–1.18)	46.00	48.68	0.764	1.11 (0.54–2.28)
rs16861194	08.63	09.05	0.823	1.05 (0.66–1.68)	00.00	07.45	–	N/A
rs6773957	49.53	51.50	0.571	1.08 (0.82–1.42)	41.38	46.94	0.498	1.25 (0.65–2.42)
rs2241767	19.82	15.79	0.125	0.76 (0.53–1.08)	24.14	19.39	0.484	0.76 (0.35–1.65)

Italicface indicates statistical significance

^a^Percent minor allele carriers

### Identification of *ADIPOQ* haplotypes associated with RPL

**Fig. 1:**
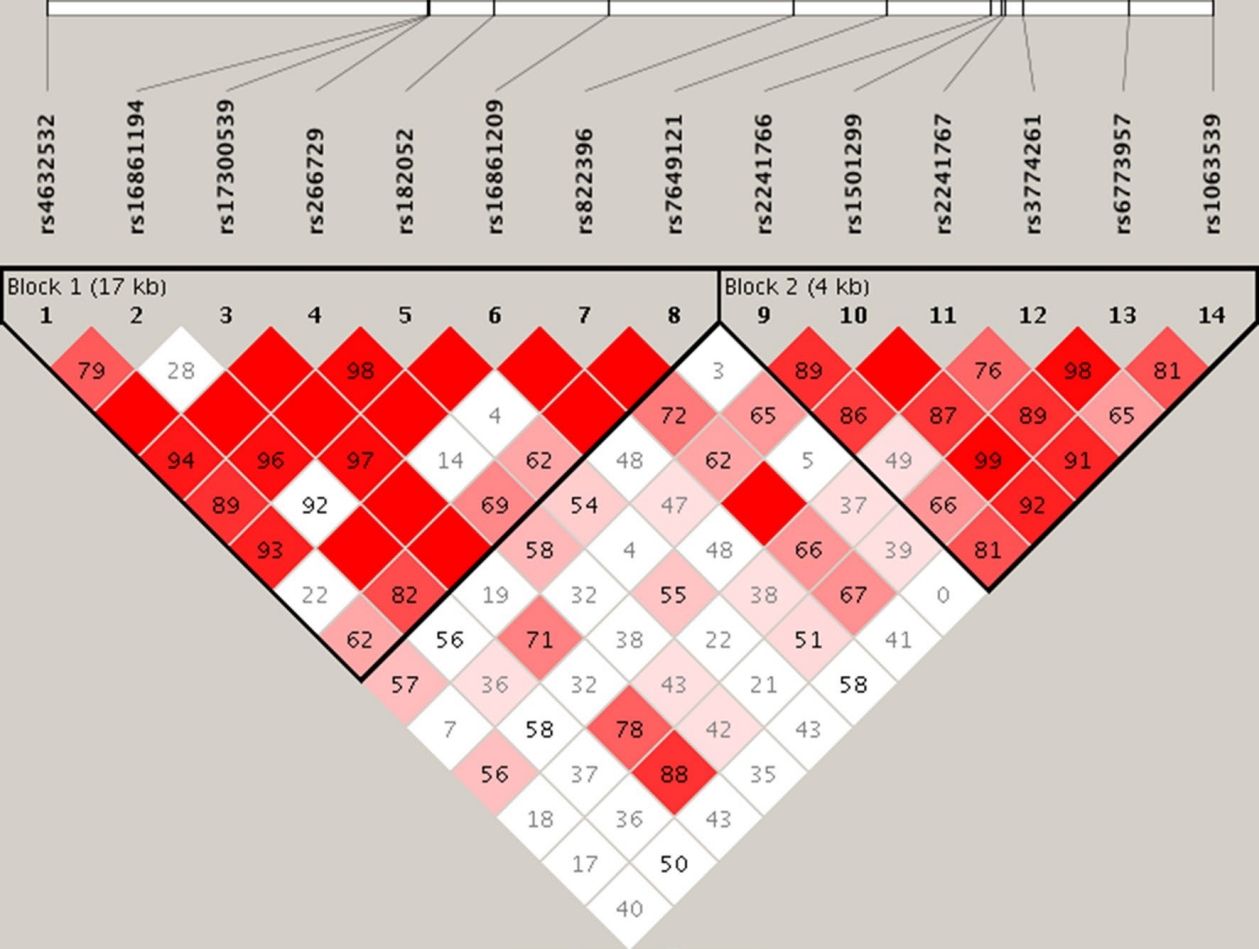
Haploview plot of *ADIPOQ* SNP analyzed. The relative positions of *ADIPOQ* SNPs (Build 37.3) are displayed, along with the basic gene structure, above the Haploview diagram. The relative LD between pairs of *ADIPOQ* SNPs is color-indicated. This was based on D’, i.e. normalized linkage disequilibrium measure or D divided by the theoretical maximum for the observed allele frequencies, multiplied by 100. Values close to zero indicate no LD, while values approaching 100 indicate full LD. The red colored square represents varying degrees of LD < 1 and LOD (logarithm of odds) > 2 scores; darker shades indicating stronger LD

The interaction between tested *ADIPOQ* variants, and their mode of inheritance in women with RPL and control women was next evaluated. The interaction between pair of SNP visualized by Haploview (Fig. 1). Haploview analysis showed marked LD among the tested *ADIPOQ* variants (Fig. 1), and defined two haploblocks. Haploblock 1 spanned 17 kb, and contained rs4632532, rs16861194, rs17300539, rs266729, rs182052, rs16861209, rs822396, and rs7649121, while Haploblock 2 spanned 4 kb, and contained rs2241766, rs1501299, rs2241767, rs3774261, rs6773957, and rs1063539.

“Common haplotype” was defined as the haplotype with frequencies > 2% of the total haplotypes. Within Haploblock 1, 94.8% of 8-locus haplotype diversity was captured by 9 of the possible 256 haplotypes. Higher frequencies of CAGGACAT (*P *= 5.1 × 10^−3^) and TAACGAAA (*P *= 0.012), and reduced frequency of TAGCGCAA (*P *= 7.6 × 10^−5^) haplotypes were seen in RPL cases, suggesting RPL-susceptible and RPL-protective aspect of these haplotypes, respectively. The frequencies of remaining haplotypes in Haploblock 1, and all haplotypes in Haploblock 2 were comparable between women with RPL and control women (Table 5).

**Table 5:** Haplotype frequencies across 14 *ADIPOQ* SNPs analyzed^a^

Haplotype^b^		Frequency	Case:control frequency	χ^2^	*P*
Block 1	*T A G C G C A A*	*0.433*	*0.369; 0.499*	*15.64*	7.6 × 10^−5^
	*C* *A G* *G* *A* *C A* *T*	*0.120*	*0.149; 0.089*	*7.86*	*5.1* *×* *10*^*−3*^
	T A G C G C G A	0.086	0.082; 0.091	0.19	0.661
	C G G C A C A A	0.068	0.076; 0.059	1.01	0.314
	*T A* *A* *C G* *A* *A A*	*0.067*	*0.087; 0.046*	*6.27*	*0.012*
	C A G G A C G A	0.048	0.053; 0.043	0.52	0.471
	T A G C G A A A	0.046	0.054; 0.038	1.23	0.268
	C A G G A C A A	0.042	0.051; 0.032	2.14	0.144
	T A G C G C A T	0.038	0.037; 0.040	0.04	0.837
Block 2	T C A G A G	0.445	0.475; 0.414	3.71	0.054
	T A A A G G	0.291	0.295; 0.287	0.06	0.801
	G C G A G C	0.125	0.119; 0.132	0.36	0.546
	T C A A G G	0.029	0.023; 0.035	1.40	0.236
	G C A G A G	0.021	0.015; 0.026	1.43	0.231

Italicface indicates statistical significance

^a^*ADIPOQ* Block 1: rs4632532- rs16861194- rs17300539- rs266729- rs182052- rs16861209- rs822396- rs7649121, Block 2: rs2241766- rs1501299- rs2241767- rs3774261- rs6773957- rs1063539 haplotypes

^b^Underlined indicates minor allele

## Discussion

### Overview of the association of *ADIPOQ* SNPs with RPL

*ADIPOQ* is a highly polymorphic gene (https://www.ncbi.nlm.nih.gov/gene/9370), and 2376 variants were identified, some of which modulate circulating adiponectin concentrations [18, 23, 25]. We investigated the association between RPL and variants in the intergenic, 5′-near gene, promoter, introns, exons, and 3′-UTR regions of *ADIPOQ* gene. Among the 14 tested variants, significant association with RPL risk was seen with intergenic (rs4632532), intronic (rs7649121, rs2241767), and exonic (rs2241766, rs1501299) *ADIPOQ* variants. This is the first report that identified these *ADIPOQ* variants as RPL at-risk loci, further supporting a role for adiponectin in the pathogenesis of RPL.

### Significance of the findings

The association between (exonic) rs1501299 (+ 276G > T) and RPL seen here was in apparent disagreement with a study on North Indian women, which reported marginal association of the + 276G > T variant (*P *= 0.0360) with reduced risk of RPL [16]. This discrepancy is likely attributed to differences in ethnic background of study participants, study selection criteria and genotyping conditions. The rs1501299 (+ 276G > T) variant was associated with altered adiponectin serum levels [26], and with hypertensive disorder complicating pregnancy (HDCP) in Chinese subjects [27].

In addition to rs1501299, rs266729 was associated with GDM in Polish [22] and Bulgarian [20], but not Brazilian women [21]; its minor (G) allele conferring protection against GDM [20]. Functionally, rs266729 was linked with altered adiponectin levels in Caucasians [17, 28], which was attributed to the capacity of its minor (G) allele to destroy Sp1 transcription factor DNA binding in the *ADIPOQ* promoter region [29].

*ADIPOQ* rs7649121 was linked with reduced risk of coronary heart disease in Chinese [30]. In addition, r rs17300539 was reportedly associated with reduced adiponectin levels in white women [31], and in T2DM patients [32]. Our study is the first to demonstrate positive association between rs7649121 and rs17300539 and RPL. On the other hand, rs2241767 was negatively associated with RPL in our cohort. This variant was associated with T2DM in Tunisian Arabs [33], and with insulin resistance in Mexican Americans [34].

*ADIPOQ* rs2241766 variant is present in exon 2, and its functional attributes are not understood, although it does not alteration of target amino acid sequence. It was reported that the carriage of rs2241766 is associated with altered mRNA splicing and/or stability [26]. While a number of studies documented association of (exonic) rs2241766 variant with pregnancy complications [21, 35], including PCOS in Brazilian [21], but not Chinese [36] women, it was not associated with RPL in our cohort. This was in agreement with the earlier study on non-obese North Indian women [16], which documented lack of association of rs2241766 with RPL.

### Obesity as risk factor of RPL: adiponectin connection

Obesity is a major contributor to infertility, and is associated with reduced success of assisted reproductive technologies [4, 37]. Given the association of reduced adiponectin to obesity and insulin resistance, a role for adiponectin in promoting successful pregnancy was suggested [14, 15]. There is a solid link between adiposity, obesity, insulin resistance and RPL [38]. Obesity was consistently associated with conditions linked with poor pregnancy outcome, including idiopathic RPL [3, 38].

Adiponectin was proposed to constitute an at-risk modulator of RPL. An earlier systematic review involving 24,738 women, concluded that obesity increases the risk of sporadic miscarriage [37], and was attributed to reduced adiponectin secretion [39, 40]. By controlling steroidogenesis and expression of genes involved in ovulation [13, 41], and induction of metabolic changes linked with adipose tissues dysfunction, particularly in genetically predisposed women [42], a central role of adiponectin in pregnancy outcome was established. In our hands, RPL-susceptible and—protective *ADIPO* SNP and haplotypes were identified, when unselected RPL cases were compared to control women, and also when RPL cases were stratified according to obesity.

Furthermore, we demonstrated that rs16861209, rs182052, and rs7649121 amplify RPL risk in non-obese women. This was in contrast to rs1063539 which was negatively associated with RPL, and presumably having a protective effect on pregnancy. On the other side, in obese subjects, rs4632532, rs182052, and rs7649121 were linked with increased RPL susceptibility, while rs2241766 was generally protective of RPL. This underscores the need for controlling for modifiable covariates in genetic association studies.

### Study strengths and shortcomings

The strength of our study is that it is sufficiently powered, and that RPL cases and control women were matched according to ethnicity, which reduces the problems of ethnic differences inherent in genetic association studies. Another strength is controlling for potential covariates. Our study has also some shortcomings. We could not measure serum adiponectin levels in cases and control women, which did not allow for addressing the functionality of this association, thus could not ascertain genotype–phenotype correlations. Another limitation lies in its retrospective nature, which prompts speculation on cause-effect relationship.

## Conclusion

We demonstrated association of *ADIPOQ* rs4632532, and rs2241766 with RPL in obese women, and rs1063539 and rs16861209 in non-obese women, and both rs182052 and rs7649121 independent of BMI changes. Future studies on additional *ADIPOQ* variants, and populations of related and distant ethnic origin are needed to support, or rule out association of *ADIPOQ* variants with altered adiponectin secretion and risk of RPL.

## Abbreviations

*ADIPOQ*: adiponectin gene
BP: blood pressure
CI: confidence intervals
GDM: gestational diabetes mellitus
HWE: Hardy–Weinberg equilibrium
LD: linkage disequilibrium
MAF: minor allele frequency
OR: odds ratios
PCOS: polycystic ovarian syndrome
PE: preeclampsia
RPL: recurrent pregnancy loss
SNP: single nucleotide polymorphisms

## References

[1] Practice Committee of American Society for Reproductive Medicine (2013). Definitions of infertility and recurrent pregnancy loss: a committee opinion. Fertil Steril.

[2] Tang AW, Quenby S (2010). Recent thoughts on management and prevention of recurrent early pregnancy loss. Curr Opin Obstet Gynecol.

[3] Lashen H, Fear K, Sturdee DW (2004). Obesity is associated with increased risk of first trimester and recurrent miscarriage: matched case-control study. Hum Reprod.

[4] Sugiura-Ogasawara M (2015). Recurrent pregnancy loss and obesity. Best Pract Res Clin Obstet Gynaecol.

[5] Hyde KJ, Schust DJ (2015). Genetic considerations in recurrent pregnancy loss. Cold Spring Harb Perspect Med.

[6] Mathew H, Castracane VD, Mantzoros C (2017). Adipose tissue and reproductive health. Metabolism.

[7] Ingram KH, Hunter GR, James JF, Gower BA (2017). Central fat accretion and insulin sensitivity: differential relationships in parous and nulliparous women. Int J Obes (Lond).

[8] Bao W, Baecker A, Song Y, Kiely M, Liu S, Zhang C (2015). Adipokine levels during the first or early second trimester of pregnancy and subsequent risk of gestational diabetes mellitus: a systematic review. Metabolism.

[9] Blüher M, Mantzoros CS (2015). From leptin to other adipokines in health and disease: facts and expectations at the beginning of the 21st century. Metabolism.

[10] Baviera G, Corrado F, Dugo C, Cannata ML, Russo S, Rosario D (2007). Midtrimester amniotic fluid adiponectin in normal pregnancy. Clin Chem.

[11] Mazaki-Tovi S, Romero R, Vaisbuch E (2010). Adiponectin in amniotic fluid in normal pregnancy, spontaneous labor at term, and preterm labor: a novel association with intra-amniotic infection/inflammation. J Matern Fetal Neonatal Med.

[12] Angelidis G, Dafopoulos K, Messini CI (2013). The emerging roles of adiponectin in female reproductive system associated disorders and pregnancy. Reprod Sci.

[13] Palin M-C, Bourdignon VV, Murphy BD (2012). Adiponectin and the control of female reproductive functions. Vitam Horm.

[14] Cortelazzi D, Corbetta S, Ronzoni S (2007). Maternal and foetal resistin and adiponectin concentrations in normal and complicated pregnancies. Clin Endocrinol (Oxf).

[15] Kawwass JF, Summer R, Kallen CB (2015). Direct effects of leptin and adiponectin on peripheral reproductive tissues: a critical review. Mol Hum Reprod.

[16] Verma PK, Prakash S, Parveen F, Faridi RM, Agrawal S (2012). Genetic association of adipokine and UCP2 polymorphism with recurrent miscarriage among non-obese women. Reprod Bio Med Online.

[17] Heid IM, Wagner SA, Gohlke H (2006). Genetic architecture of the APM1 gene and its influence on adiponectin plasma levels and parameters of the metabolic syndrome in 1727 healthy Caucasians. Diabetes.

[18] Chung HK, Chae JS, Hyun YJ (2009). Influence of adiponectin gene polymorphisms on adiponectin level and insulin resistance index in response to dietary intervention in overweight-obese patients with impaired fasting glucose or newly diagnosed type 2 diabetes. Diabetes Care.

[19] Menzaghi C, Ercolino T, Salvemini L (2004). Multigenic control of serum adiponectin levels: evidence for a role of the APM1 gene and a locus on 14q13. Physiol Genom.

[20] Beltcheva O, Boyadzhieva M, Angelova O, Mitev V, Kaneva R, Atanasova I (2014). The rs266729 single-nucleotide polymorphism in the adiponectin gene shows association with gestational diabetes. Arch Gynecol Obstet.

[21] Machado JS, Cavalli RC, Sandrim VC (2012). Study of polymorphisms of the adiponectin gene in women with gestational hypertension and preeclampsia. Pregnancy Hypertens.

[22] Pawlik A, Teler J, Maciejewska A, Sawczuk M, Safranow K, Dziedziejko V (2017). Adiponectin and leptin gene polymorphisms in women with gestational diabetes mellitus. J Assist Reprod Genet.

[23] Park JW, Park J, Jee SH (2011). ADIPOQ gene variants associated with susceptibility to obesity and low serum adiponectin levels in healthy Koreans. Epidemiol Health.

[24] Saarela T, Hiltunen M, Helisalmi S, Heinonen S, Laakso M (2006). Adiponectin gene haplotype is associated with preeclampsia. Genet Test..

[25] Peters KE, Beilby J, Cadby G, Warrington NM, Bruce DG, Davis WA, Davis TM, Wiltshire S, Knuiman M, McQuillan BM, Palmer LJ, Thompson PL, Hung J (2013). A comprehensive investigation of variants in genes encoding adiponectin(ADIPOQ) and its receptors (ADIPOR1/R2), and their association with serum adiponectin, type 2 diabetes, insulin resistance and the metabolic syndrome. BMC Med Genet.

[26] Ranjzad F, Mahmoudi T, Irani Shemirani A, Mahban A, Nikzamir A, Vahedi M, Ashrafi M, Gourabi H (2012). A common variant in the adiponectin gene and polycystic ovary syndrome risk. Mol Biol Rep.

[27] Wang Y, Liu RX, Liu H (2015). Association of adiponectin gene polymorphisms with hypertensive disorder complicating pregnancy and disorders of lipid metabolism. Genet Mol Res.

[28] Vasseur F, Helbecque N, Lobbens S (2005). Hypoadiponectinaemia and high risk of type 2 diabetes are associated with adiponectin-encoding (ACDC) gene promoter variants in morbid obesity: evidence for a role of ACDC in diabesity. Diabetologia.

[29] Zhang D, Ma J, Brismar K, Efendic S, Gu HF (2009). A single nucleotide polymorphism alters the sequence of SP1 binding site in the adiponectin promoter region and is associated with diabetic nephropathy among type 1 diabetic patients in the genetics of kidneys in diabetes study. J Diab Compl.

[30] Liang C, Yawei X, Qinwan W, Jingying Z, Aihong M, Yanqing C (2017). Association of *ADIPOQ* single-nucleotide polymorphisms and smoking interaction with the risk of coronary heart disease in Chinese Han population. Clin Exp Hypertens.

[31] Cohen SS, Gammon MD, North KE (2011). *ADIPOQ*, ADIPOR1, and ADIPOR2 polymorphisms in relation to serum adiponectin levels and BMI in black and white women. Obesity.

[32] Siitonen N, Pulkkinen L, Lindström J (2011). Association of ADIPOQ gene variants with body weight, type 2 diabetes and serum adiponectin concentrations: the finnish diabetes prevention study. BMC Med Genet.

[33] Mtiraoui N, Ezzidi I, Turki A, Chaieb A, Mahjoub T, Almawi WY (2012). Single-nucleotide polymorphisms and haplotypes in the adiponectin gene contribute to the genetic risk for type 2 diabetes in Tunisian Arabs. Diabetes Res Clin Pract.

[34] Richardson DK, Schneider J, Fourcaudot MJ (2006). Association between variants in the genes for adiponectin and its receptors with insulin resistance syndrome (IRS)-related phenotypes in Mexican Americans. Diabetologia.

[35] Ye Y, Pu D, Liu J, Li F, Cui Y, Wu J (2013). Adiponectin gene polymorphisms may not be associated with idiopathic premature ovarian failure. Gene.

[36] Zhang W, Wei D, Sun X, Li J, Yu X (2014). Family-based analysis of adiponectin gene polymorphisms in Chinese Han polycystic ovary syndrome. Fertil Steril.

[37] Boots C, Stephenson MD (2011). Does obesity increase the risk of miscarriage in spontaneous conception: a systematic review. Semin Reprod Med.

[38] Khandouzi M, Deka M (2014). The role of adiponectin in human pregnancy. Int J Res Engin Technol.

[39] Cikos S, Burkus J, Bukovska A, Fabian D, Rehak P, Koppel J (2010). Expression of adiponectin receptors and effects of adiponectin isoforms in mouse pre-implantation embryos. Hum Reprod.

[40] Kim ST, Marquard K, Stephens S, Louden E, Allsworth J, Moley KH (2011). Adiponectin and adiponectin receptors in the mouse preimplantation embryo and uterus. Hum Reprod.

[41] Richards JS, Liu Z, Kawai T (2012). Adiponectin and its receptors modulate granulosa cell and cumulus cell functions, fertility and early embryo development in the mouse and human. Fertil Steril.

[42] Hivert MF, Scholtens DM, Allard C (2017). Genetic determinants of adiponectin regulation revealed by pregnancy. Obesity.

